# Normalizing the Supernormal: The Formation of the “Gesellschaft Für Psychologische Forschung” (“Society for Psychological Research”), c. 1886–1890

**DOI:** 10.1002/jhbs.21577

**Published:** 2012-11-20

**Authors:** Andreas Sommer

## Abstract

This paper traces the formation of the German “Gesellschaft für psychologische Forschung” (“Society for Psychological Research”), whose constitutive branches in Munich and Berlin were originally founded as inlets for alternatives to Wundtian experimental psychology from France and England, that is, experimental researches into hypnotism and alleged supernormal phenomena. By utilizing the career trajectories of Max Dessoir and Albert von Schrenck-Notzing as founding members of the “Gesellschaft,” this paper aims to open up novel perspectives regarding extra-scientific factors involved in historically determining the epistemological and methodological boundaries of nascent psychology in Germany.

The establishment of Wilhelm Wundt's laboratory of experimental psychology in Leipzig in 1879 and foundation of his journal *Philosophische Studien* in 1881 ultimately signified the institutionalization of the “new psychology” in Germany. A field hitherto little explored by historians is the German reception of alternatives to physiological psychology, notably English and French psychical research and studies in hypnotism, as negotiated at the International Congresses of Psychology from 1889 to 1909. A small but visible forum for psychologists dissatisfied with the Wundtian dominance of nascent German psychology serving as a conduit for French and English strands of experimental psychology was the “Gesellschaft für psychologische Forschung” (“Society for Psychological Research”), with the philosopher-psychologist Max Dessoir (1867–1947) as the secretary of its Berlin section and the physician Albert von Schrenck-Notzing (1862–1929) leading the Munich branch. The “Gesellschaft,” founded in November 1890, was an amalgamation of two previously existing associations, the “Psychologische Gesellschaft” (“Psychological Society”) in Munich, cofounded by Schrenck-Notzing, and the “Gesellschaft für Experimental-Psychologie” (“Society of Experimental Psychology”) in Berlin under the leadership of Max Dessoir.

In her groundbreaking study of *fin-de-siècle* German occultism, Corinna Treitel (2004) noted that

although historians have been strangely reluctant to examine these two developments together [i.e. the co-emergence of psychical research and academic psychology], there is much evidence to suggest that no history of German psychology will be complete without such an examination. (p. 45)

Following Treitel's hint, this paper offers an expansion of Heather Wolffram's (2009) thesis of German psychical research as a “border science,” that is, a field whose leading proponents—Schrenck and Dessoir—were careful not to trespass into the territories of fledgling academic psychology from its very inception. While the “boundary” status certainly applies to post-World War I parapsychology in Germany and elsewhere, it is obvious that Schrenck and Dessoir had initially intended to challenge, or at least broaden, the narrow scope of fledgling German psychology. By acting as conduits for French and English strands of experimental psychology, they in fact closely followed the example of the founder of American psychology, William James.

Historians of psychology remember Max Dessoir, a student of Wilhelm Dilthey, Adolf E. Fick, and Hermann Munk, as the author of the first comprehensive bibliography of hypnotism in German (Dessoir, 1888a), for publishing an early influential study of the psychology of dissociation (Dessoir, 1890b), and for his histories of psychology (Dessoir, 1894, 1911, 1912). Dessoir is also known for his coinage of the term “Parapsychologie” as a young man in the late 1880s in an attempt to delineate the scientific study of a certain class of “abnormal,” though not necessarily pathological mental phenomena. The name of Albert von Schrenck-Notzing, on the contrary, is now largely obscure to historians of psychology and medicine. The violent controversies around his studies of the alleged physical phenomena of mediumship, which he published from 1914 onward, still appear to blur Schrenck's historical significance in hypnotism and sexology about two decades previously.^1^ However, up to his becoming the doyen of early-twentieth-century German psychical research, Schrenck, who had studied hypnotism with Freud under Bernheim in the late 1880s, was an internationally recognized pioneer in the empirical and clinical study of hypnotism and sexual deviations and a sought-after forensic expert.

Utilizing the history of Dessoir's and Schrenck-Notzing's involvement in shaping the new science of psychology, this article traces the formation of the “Gesellschaft für Psychologische Forschung” to identify German conduits of French and English strands of experimental psychology as an alternative to Wundtian physiological psychology and its offshoots. Moreover, it argues for the importance of extra-scientific factors that have resulted in the segregation of certain controversial research questions from the agenda of academic psychology, which some of the founding figures of modern psychology in Europe and America had considered legitimate fields of scientific investigation.

## Wilhelm Hübbe-Schleiden, Carl du Prel, and the *Sphinx*

Prior to their fusion in 1890, both the Munich “Psychologische Gesellschaft” (MPG) and the Berlin “Gesellschaft für Experimental-Psychologie” (GEP) were founded as psychical research societies similar to the Society for Psychical Research (SPR) in England, whose research program—studies of telepathy, apparitional experiences, mediumship, and hypnotism—they attempted to emulate.^2^ The foundation of the MPG in October 1886 was preceded by the inception of the journal *Sphinx* in January of the same year by the jurist, colonial politician, and theosophist Wilhelm Hübbe-Schleiden (1846–1916), a central figure in the sphere of German-language intellectuals interested in the “occult.” A previously existing journal concerned with alleged psychic phenomena had been *Psychische Studien*, founded by the Russian councilor of the state Alexandr Aksakov (1832–1903) in 1874 and edited by the excommunicated theologian Gregor Constantin Wittig (1834–1908).^3^ In a letter to Eduard von Hartmann (1842–1906), whom he invited to collaborate in the upcoming journal, Hübbe-Schleiden had justified the envisaged foundation of the *Sphinx* by stressing the need for an alternative to *Psychische Studien*, which was narrowly focused on debates around spiritism.

The publication agenda of the *Sphinx* was tailored around the promotion of the “transcendental psychology” of Carl du Prel (1839–1899) as outlined in his *Philosophie der Mystik* (du Prel, 1885a).^4^ Shortly after receiving a PhD degree in philosophy from Tübingen University, du Prel had launched his career as a private scholar by becoming Eduard von Hartmann's philosophical henchman, propagating the latter's *Philosophy of the Unconscious* (1869) and eloquently defending it from critics (du Prel, 1870, 1872a, 1872b), which Hartmann rewarded with his long-lasting support. However, from about 1878, du Prel started to find his own philosophical legs and began, much to the dislike of his former mentor, transforming parts of Hartmann's philosophical synthesis into a “metaphysical individualism.” Based on studies of the psychology of dreams (du Prel, 1869, 1882c), epistemological deductions from Darwinism and astronomy (du Prel, 1880), the literature of animal magnetism and somnambulism (du Prel, 1882a, 1884), and the ideas of the Austrian philosopher Lazar von Hellenbach (1827–1887; see, e.g., Hellenbach, 1876, 1885), du Prel developed his “monistic” theory of the “transcendental subject,” which he equated with the individual unconscious mind. For du Prel, the “transcendental subject” was both the thinking and organizing principles in man, which preexisted as well as survived the physical organism, thus offering a rational and secularized basis for a belief in life after death.^5^ Hartmann, who rejected ontologies of a personal unconscious and its survival of bodily death, criticized du Prel for what he thought were strongly exaggerated and premature deductions from anecdotal and outdated reports of spontaneous and induced somnambulism, which served as empirical cornerstones of du Prel's ideas. Rather than offering a key to the individual unconscious and its survival of death, Hartmann held that somnambulism was an intrinsically pathological state (Hartmann, 1886, chapter 12).

Du Prel's “monistic” transcendental psychology dominated the *Sphinx* up to the early 1890s. Fittingly, from its foundation in January 1886 to a shift in focus toward theosophy in 1891, the journal's subtitle was “Monthly for the historical and experimental foundation of the super-sensory worldview on the basis of monism.”^6^ The first issue in January 1886 was inaugurated by du Prel's article “Monistic psychology” (du Prel, 1886), which had been the title of the concluding section of his *Philosophy of Mysticism* and that of his second book, *Die monistische Seelenlehre* (du Prel, 1888), in which the *Sphinx* article was to be incorporated.^7^ The considerable impact of du Prel's ideas on the initial scope of the journal was also visible in a “Preface and appeal to readers” in the first issue. There, *Sphinx* editor Hübbe-Schleiden identified the establishment of the thinking and organizing function of the unconscious as the core features of a “monistic” transcendental psychology and identified du Prel's research agenda—the integrative investigation of mesmerism, hypnotism, clairvoyance, and mediumship—as that of the new journal. Hinting to German experimental psychology's neglect to investigate these areas, Hübbe echoed a concern previously expressed by du Prel:

A science that by choice relinquishes these aids for an explanation of man artificially impedes its task, which is difficult enough, while it only occurs to the researcher in the field of the super-sensory that, in fact, even the every-day phenomena of mental life become comprehensible through such transcendental science only. (1886, p. II)

It was owing to the purpose of establishing du Prel's transcendental psychology, whose empirical foundations were research into dreams, mesmerism, somnambulism, and hypnotism, that the *Sphinx* became one of the most important—if not the most important—early German periodicals serving as a conduit for the latest works in hypnotism from France and England. For instance, the journal published German translations of articles by authors such as Hippolyte Bernheim (1887, 1891), Ambroise-Auguste Liébeault (1888, 1891), and Frederic W. H. Myers (1887). More significantly, both Dessoir and Schrenck regularly reviewed or summarized the latest works by authors such as Bernheim, Beaunis, Binet, Gurney, and other foreign pioneers in hypnotism research in the *Sphinx* (e.g., Dessoir, 1887a,^8^ 1888d; [Schrenck-]Notzing, 1888b, 1889). Dessoir published a preliminary version of his acclaimed bibliography of hypnotism in the *Sphinx* (Dessoir, 1887d), and his surviving letters to Hübbe suggest that the latter, by regularly providing the impecunious student with works on hypnotism and psychology from his own vast library and by arranging review copies, was a significant resource for Dessoir's early literary input. Schrenck, whose career as one of the foremost early hypnotists in Germany swiftly launched after obtaining his MD degree in 1888 with a thesis on hypnotherapy, likewise contributed a considerable number of reviews and notes regarding hypnotism to the *Sphinx*, for which he also translated an article on hypnotherapy by Bernheim (1891).^9^

Shortly after the foundation of the “Münchner Psychologische Gesellschaft” in October 1886 by du Prel, Hübbe, Schrenck, and others, the *Sphinx* was to become its publication organ.^10^ With Adolf Bayersdorfer, conservator of the “Alte Pinakothek” (an important art center in Munich), as president, Hübbe-Schleiden as vice president, and the MD candidate Schrenck-Notzing as secretary, the constitution of the society defined its objects thus:

The purpose of the society is to facilitate among its members the study of psychology through scientific lectures, discussion evenings, experiments and social gatherings, and particularly, to its best endeavors, to promote the scientific recognition of facts from the area of the transcendental. (§1, constitution of the “Psychologische Gesellschaft,” cited in Kaiser, 2008, p. 255)

The January 1887 issue of the *Sphinx* incorporated the “Program of the Psychological Society in Munich” written by du Prel (1887), outlining his definition and suggested scope of a scientific psychology as previously proposed in his *Philosophy of Mysticism*, and which the newly founded MPG should adhere to.^11^ Joining the ranks of contemporary philosophers decrying a German “psychology without a soul’, du Prel observed that in order to liberate psychology from its constricting materialistic epistemology, it was the psychologist's foremost task to “contrast the undeniable influence of the physical on the mental with the influence of the mental on the physical, and to emphasize those psychical functions that vouch for their independence from the physical body in themselves” (p. 32). Du Prel argued that dreams and altered states such as somnambulism and hypnotic trance were superior to the normal waking state as fields of psychological investigation, since they were conducive to an induction, quasi-isolation, and thus systematic exploration of transcendental functions that could throw light on the very nature of the psyche. Referring to experiments by authors such as Bernheim and Henri Beaunis, which had reported the hypnotic induction of blisters, stigmata of specific shapes, and other vasomotor anomalies, du Prel inferred that modern hypnotism had already refuted medical materialism by proving the dependency of even unconscious and involuntary bodily functions on the mental, for “this obviously points to an identity of the thinking and the organizing principle in us, and this fact among many others is already sufficient for the foundation of a monistic doctrine of the soul” (p. 33).

Arguing that the “transcendental-psychological” phenomena of somnambulism had already been familiar in antiquity and were thus merely rediscovered by Mesmer, Puységur, and their followers (p. 34), du Prel stressed that thought-reading, clairvoyance, action at a distance, and other phenomena tabooed by orthodox science had been reported throughout history and could therefore not simply be dismissed without examination. Complaining that these transcendental functions of the soul had been monopolized by institutionalized religion since antiquity, du Prel demanded that psychologists should study these phenomena experimentally rather than dismiss them *a priori*.

In accordance with his previous critiques of modern science's loss of synthetic perspective in the course of disciplinary specialization (du Prel, 1881, 1882b), du Prel's MPG program predicted that there was hardly a profession that would fail to profit from an integrative study of transcendental psychology, especially naming philosophers, cultural historians, physicians, philologists, educators, psychiatrists, theologians, lawyers, and artists as potential beneficiaries (p. 35). He reinstated that, unlike official science, the MPG was dedicated to investigate rather than reject off-hand “the little investigated aspects of mental life” and that “experiment must bring clarity even upon the most extreme phenomena of this kind—such as those which have much been talked about under the name of spiritism” (p. 35). Implicitly referring to previous denunciations of psychical research by psychologists such as Wilhelm Wundt (1879) and Wilhelm Preyer (1878, 1886a, 1886b), du Prel demanded:

It is clear enough that spiritism will not be banished by mere exclamations of authority from the standpoint of preconceived systems; he who wants to abolish it depends on its investigation just as much as he who wants to promote it.

concluding that if there was a science that could ill afford premature theoretical commitment by willfully excluding the study of phenomena apt to fundamentally outstrip a reductive physiological framework, it was psychology (p. 36).

## Max Dessoir and the foundation of the “Gesellschaft für Experimental-Psychologie”

While *Sphinx* editor Wilhelm Hübbe-Schleiden was directly involved in the formation of the MPG as a founding member and vice president, he was, though less immediate, also instrumental in the foundation of the GEP by Dessoir, the art historian Friedrich Goeler von Ravensburg, the police superintendent Johannes von Manteuffel, and the bookseller and editor Hans Natge in January 1888. This is exemplified in his friendship with and support of the young Max Dessoir, who, still a student, was a regular *Sphinx* contributor before and after the foundation of the GEP. Dessoir's letters to Hübbe^12^ suggest a greater indebtedness to the theosophist than Dessoir's memoirs would later reveal, which acknowledge Hübbe's “friendly support” but briefly (Dessoir, 1947a, p. 125). Apart from supplying young Dessoir with the latest literature in psychology and hypnotism, Hübbe paid him generous fees for articles, offered occasional career advice, and arranged important contacts. In fact, it was Hübbe who introduced Dessoir to Goeler von Ravensburg, Hans Natge, and other founding and early members of the GEP, several of whom had contacted Hübbe by letter, who then referred them to Dessoir as his “man in Berlin.”

Dessoir was among those *Sphinx* collaborators who, though initially supporting the project of an empirically based transcendental psychology in principle, were critical of du Prel's approach. As early as 1886, he expressed his frustration with du Prel, who, Dessoir complained to Hübbe, “wishes to base a general metaphysical inference on 2 facts.”^13^ By this time, another important influence in young Dessoir's life was developing, namely, his friendship with Eduard von Hartmann, who was also to become a member of the GEP and who was now among du Prel's most vocal critics.^14^ In 1887, Dessoir repeatedly began to press for an “inner change” of the *Sphinx* in his letters to Hübbe, which he believed was “absolutely necessary.”^15^ When Hübbe considered parting ways with his current publisher to have the journal print privately, Dessoir strongly advised against it, asking Hübbe to

rest assured that I will find means and ways to help us get going. Moreover, Hartm.[ann] says that he will gladly do anything he can; also Goeler, both of whom ask me to pass on their regards. However, the content must change as well. Don't resent me, but not even the healthiest stomach can eventually tolerate those du Prelian tapeworms (which is not *my* feeling *alone*).^16^

Hartmann's and Dessoir's concerns were also shared by members of the MPG, whose young secretary and former pupil of du Prel, Schrenck-Notzing, also favored the more cautious research policy employed by the SPR in England and William James in America, which was to empirically establish the facts in question beyond reasonable doubt first and then only to ponder their theoretical implications. Young men like Dessoir and Schrenck felt that the German leaders of scientific psychology, who had either ignored psychical research, or, like Wundt and Preyer, publicly attacked and demarcated it from physiological psychology as the only legitimate experimental science of the mind, could never be won over if German psychical research was associated with the lofty speculations of du Prel rather than the empirical rigor typical of the SPR work.^17^

The formation of the Berlin “Gesellschaft für Experimental-Psychologie”^18^ in January 1888 by Dessoir, Goeler von Ravensburg, Manteuffel, and Natge can thus be viewed as a response to wider concerns regarding du Prel's metaphysical agenda, which threatened to become a popular—and overly radical—alternative to conservative Wundtian experimental psychology. This was reflected in the research program of the GEP, which was also published in the *Sphinx* (Goeler von Ravensburg & Dessoir, 1888): Naming the SPR, its American sister-society, the French “Société de Psychologie Physiologique” (SPP), and the MPG as models, the expressed focus of the GEP was on the replication of results hitherto published by representatives of these associations. Emphasizing the difference of French and English strands of experimental psychology from German physiological psychology as well as from introspective psychology, a methodological pluralism was advocated, which was to employ experimental research and statistics as well as ethnological and historical approaches (p. 299). However, despite overlaps in the research programs of the GEP and MPG, the former contained an important caveat signifying a clear rejection of du Prel's agenda. For although the envisaged work of the GEP was expected to yield important insights into “the nature of the human soul,” and though “the philosophical treatment, the speculative utilization of the material empirically obtained” was stated to figure among the tasks of the GEP as well, the program unambiguously identified the scientific status of such speculations:

However, this can only be a matter of individual conviction rather than of corporate views of the Society. Most of all, it must not be overlooked that all our investigations and works are to emanate from a critical verification of facts and their methodical treatment, not from philosophical views, from theories and hypotheses. (pp. 299–300)

At the time of the GEP's foundation, conflicts between Schrenck's and du Prel's camps within the MPG had become overt although they did not appear to be limited to scientific disagreements alone. In 1888, MPG president Bayersdorfer apparently threatened to exclude Schrenck from the society if the latter was to follow through his intention to sue a patient who had claimed that Schrenck's hypnotic treatment had harmed him, which Bayersdorfer deemed a political fiasco (Kaiser, 2008, p. 65). However, it was du Prel rather than his former pupil Schrenck who eventually left the MPG in 1889 to establish a separate group, the Munich “Gesellschaft für Experimentalpsychologe” (“Society for Experimental Psychology”), which soon changed its name into “Gesellschaft für wissenschaftliche Psychologie” (“Society for scientific psychology”) and which was primarily concerned with philosophical speculation rather than empirical investigations. Details regarding the specific content of the conflicts, and information as to why it was du Prel rather than the much younger Schrenck who left the original MPG, remain obscure; all that is known is that subsequently du Prel would fully blame his former pupil for the schism.^19^

## Normalizing the Supernormal: Dessoir, Schrenck-Notzing, and the “Will to Conform”

Both Dessoir's and Schrenck's increasing distance from du Prel significantly correlated with their entering academia, i.e., Schrenck's MD degree in 1888, and Dessoir's PhD degree in 1889. Moreover, the schism within the MPG corresponded with a number of notable changes in the official positions of Schrenck and Dessoir regarding the “supernormal,” of which the ultimate split between Schrenck and his former mentor seemed but a symptom.

By 1887, both Schrenck and Dessoir had fully embraced the reality of telepathy as a scientifically demonstrated fact, having published own experiments replicating findings of foreign scholars (Dessoir, 1886a, 1886b, 1886c; [Schrenck-]Notzing, 1886, 1887a, 1887b, 1887c). Schrenck would still frame the spontaneous occurrence of ostensibly precognitive dreams in terms of du Prel's theory of the “transcendental subject” ([Schrenck-]Notzing, 1887d), and Dessoir (1886d), explicitly in support of du Prel's criticism of Wilhelm Preyer's rejection of telepathy in terms of “muscle-reading” (du Prel, 1885b), wrote that one needed to distinguish three causes of “thought-transference”: fraud, unconscious muscle-reading, and finally genuine telepathy, the reality of which Dessoir deemed established beyond reasonable doubt through published studies from France, England, and Munich, as well as by his own experiments with and without physical contact between the subject and the experimenter (Dessoir, 1886d, p. 258; Kurzweg, 1976, pp. 101–104).^20^ Also in 1886, Dessoir had concluded the publication of an apparently successful series of telepathy experiments by stating that “it has ceased to be a question of *proof* of the facts of super-sensory thought-transference: all that matters now is merely to ascertain the *conditions* of such transference” (Dessoir, 1886a, p. 248, original emphases). In 1887, Dessoir would still publicly admit his convictions, for instance in the German revue *Die Gegenwart*, where he published a review of Edmund Gurney's reply to Wilhelm Preyer's sweeping rejection of the work of psychical researchers in England and France, in which Gurney had politely identified Preyer's misrepresentations of the original work (Dessoir, 1887b; Gurney, 1887).

The following year, which, we remember, saw the foundation of the GEP as well as the launch of Schrenck's promising medical career, had another event in store that possibly contributed to Dessoir's change of mind regarding psychical phenomena and their reported occurrence in hypnotism, namely, Edmund Gurney's death in June 1888. It seems that Gurney, who had proposed young Dessoir as a corresponding member of the SPR in 1887,^21^ was the one figure in psychical research whom Dessoir had admired the most, which is reflected, for example, in Dessoir's review of Gurney's reply to Preyer, a devastated obituary of Gurney in the *Sphinx* (Dessoir, 1888b) and Dessoir's memoirs, were he wrote that Gurney's death caused him “great sorrow” and that their friendship was based not on their interest in psychical research alone but also on their mutual love of music (Dessoir, 1947a, p. 124).^22^ While Gurney—one of William James’ closest friends in England—represented the most accomplished work of the “positive” wing of psychical research, it is fair to say that after Gurney's death, his influence on the young Dessoir began to be substituted by that of the militantly skeptical Albert Moll, which started in 1888, when Moll joined the newly founded GEP.^23^ On January 22, 1888, Dessoir announced to Hübbe that he would like Moll to join the GEP, asking Hübbe for the latter's contact details.^24^ Shortly after Moll had accepted Dessoir's invitation to join the GEP in January 1888, Dessoir wrote to Hübbe: “Moll is a very capable man, with whom I became good friends and who will help us a lot.”^25^ A few months later, Dessoir signaled to Hübbe that it was no longer necessary to send him the latest issues of the *Revue hypnotique* as Moll was now his literary supplier.^26^

Around the same time, another important influence on young Dessoir was beginning to form, namely, his collaboration with the ethnologist Adolf Bastian (1826–1905). In July 1887, Dessoir mentioned to Hübbe: “By the way, I am pursuing a little *Voelkerpsychologie* with Bastian,” and Dessoir seems to have motivated Bastian to write an article on spiritism and ethnology in the *Sphinx* (Bastian, 1887). Bastian would also give a talk at the GEP, which was published as a part of the society's transactions (Bastian, 1890). The take-home message of Bastian's lecture, titled “On psychic observations in primitive people,” was that belief in “occult” principles signified a natural developmental stage in indigenous societies but was a regressive and intrinsically pathological phenomenon in modern civilized cultures, with detrimental effects on society. In this letter, Dessoir also proposed, probably for the first time, the term “Parapsychologie.” Suggesting a new subtitle of the *Sphinx*, Dessoir wrote to Hübbe: “What do you think: ‘Sphinx.’ Monthly for parapsychology? Παρα signifies anything that goes ‘alongside the normal’ (παρανoµια), paraps.[ychology] would thus be the doctrine of the anomalous phenomena of the soul.”^27^

Probably the most decisive factor contributing to Dessoir's modified official standpoint regarding the supernormal was the need to respond to negative publicity in 1888 and 1889. First, the involvement of GEP members (Natge, Bentivegni, and, briefly, Dessoir) in the investigation of a “poltergeist” case in Resau near Berlin dramatically backfired on the public image of the society. The episode centered around Karl Wolter, a 14-year-old peasant boy, in whose presence raps were heard, windows were smashed, and objects and foods were thrown or moved without visible causation. Under considerable involvement of the press, the case quickly turned into a witch hunt, resulting in the underage Karl Wolter's sentencing for two weeks’ prison and four weeks’ detainment for public nuisance in 1889 in the second instance. As Kurzweg's (1976, pp. 202–256) painstaking reconstruction of the events has revealed, Wolter was sentenced despite the absence of any evidence of the supposed hoax and the verdict was purely based on the lawyers’ opinion—the defenders’ suggestion to consult scientific experts on the matter was rejected—that the movement of objects without a visible cause was a scientific impossibility. A further revealing indicator for the social undesirability of the “occult” was Wolter's employment by the court conjuror Max Rößler in Berlin following Wolter's imprisonment. Rößler used the boy as an object of ridicule in his widely publicized performances, which sailed under the flag of anti-spiritist propaganda. In the court proceedings, as well as in newspaper reports, Natge, Bentivegni, and Dessoir were tacitly associated with unenlightened tendencies by being wrongly referred to as members of a spiritualist club rather than a research association.

Following the Resau controversy, the GEP's publicity was not exactly helped by an attack in early 1889 by the rising star of German experimental psychology, Hugo Münsterberg, in Freiburg. In a talk on “thought-transference,” the ambitious psychologist protested that the activities of the Berlin and Munich psychological societies would severely delay the establishment of German academic psychology because of their association with spiritualism. After fueling public fears of the “occult” as a threat to social order, Münsterberg explained that academic psychologists like himself could not be held responsible for “all those unmethodically gathered miracles, which sail under the flag of experimental psychology, just as little as astronomy is responsible if nowadays someone publishes astrological prophecies, or as chemistry if someone promises to make gold through alchemical art” (Münsterberg, 1889, p. 3) and that it was the responsibility of scientific psychologists to publicly demarcate their work from the “unscientific” investigations of the psychological societies in Berlin and Munich.

With Resau and direct attacks by representatives of academic psychology drastic measures were needed to promote the public image of the GEP and its sister-society in Munich. Thus, following the exodus of du Prel's camp from the MPG, Dessoir gave a programmatic talk to the MPG on June 27, 1889, titled “On the scope and methods of the psychological societies,” which was published on October 17, 1889, in the supplement of the *Allgemeinen Zeitung*, a widely read newspaper. The printed version of the talk roughly coincided with the publication of Dessoir's celebrated work *Das Doppel-Ich*, which Dessoir had presented to the GEP on March 12, 1889.^28^ The published text of Dessoir's MPG talk on the work of the psychological societies was a radical revision of the programs of both the GEP and MPG. The essence of Dessoir's manifesto was that hypnotism was to be considered completely dissociated from its connections to any “occult” phenomena such as telepathy or clairvoyance and that psychological automatisms were now more or less explained and understood by Dessoir's theory of the “double self.”

The publication of Dessoir's *Doppel-Ich* as the first installment of the transactions (*Schriften*) of the GEP in summer 1889 can be regarded as serving a twofold purpose: First, as an attempt at introducing Pierre Janet's and Theodule Ribot's “experimental psychopathology” to German psychology and to reconcile it with German physiological psychology. Second, by making his strictly positivistic model of dissociation as an alternative to du Prel's theory of the “transcendental subject” the trademark of the GEP, Dessoir aimed to once and for all cleanse the society from any “occult” connotations. Joining the choir of orthodox psychology, Dessoir now explained any nonphysiological interpretation of ostensible supernormal phenomena in terms of an unscientific “need to believe” that would necessarily contaminate the outcome of investigations. Accordingly, Dessoir disqualified du Prel and his camp by stating that “surely, the disjunction of a transcendental Subject from the empirical self as proposed by du Prel has hardly a higher value than that of an interpretation of certain facts due to a metaphysical need to believe” (Dessoir, 1890b, p. 30). Largely steering clear from treating supernormal phenomena in *Das Doppel-Ich*, Dessoir now explained that if thought-transference turned out to be a genuine phenomenon at all, it was perfectly possible to account for it in terms of a physical theory, that is, as a “concordance of association” (*Assoziationskonkordanz*, p. 31) of resonating brain states or other physiological correlates. Thus, Dessoir ensured that psychologists of Preyer's, Wundt's, and Münsterberg's ilk no longer needed to worry about any barefaced metaphysical implications of hypnotism, automatisms, and dissociation as emphasized by du Prel.

Dessoir's *Allgemeine Zeitung* article on the psychological societies officially cemented the new orientation of the GEP in the public mind and was the crucial step toward its fusion with the “new” MPG in the following year. This is obvious from Dessoir's characterization of the Munich Society under the leadership of Schrenck:

Whereas the works published during its first cycle of existence were more of a philosophical nature, since recently it has wholly followed the inductive mode of investigation and the more cautious proceedings of its sister societies [i.e., the SPR, ASPR, SPP and GEP] and is now continuing to work, under the aegis of Dr. von Schrenck, in the sense of current science. (1889c, p. 1)

Following this epistemic rehabilitation of the group around Schrenck, it was important to stress the difference between the “new” MPG and du Prel's association, which Dessoir referred to as a small group of men “who believe in a magnetic agent alongside suggestion, in the somnambulic faculties denied by medicine, and in spiritism in the actual sense of the word” (p. 1). Dessoir claimed that benefits of research in hypnotism and the psychology of dissociation as conducted by the GEP and MPG would involve advances in medical therapy and, for society at large, the “safety from superstitious tendencies” by scientifically illuminating psychological phenomena whose false interpretation posed a danger to public welfare (p. 2). Explicitly appealing to academic psychologists for support and cooperation, Dessoir proclaimed that it was the task of scientific psychology to disentangle the “stepchildren of official science”—mental phenomena such as hypnotism and automatisms—from their occult ballast.

Without naming du Prel, Dessoir also launched an unambiguous attack on the philosopher. For Dessoir, men such as du Prel (who had published in popular rather than academic outlets) were “people who never went through the educational mills of natural-scientific or medical studies, laymen in the worst sense of the word, armchair philosophers (*Schneckenhausphilosophen*) who have constructed a pseudo-psychology (*Zopfpsychologie*) out of their fossil systems” (Dessoir, 1889c, p. 2). For Dessoir, people such as du Prel, whom he accused to consider spiritism as an “experimental religion,” had no room in truly scientific psychological societies such as the GEP and Schrenck's MPG.

In October 1889, du Prel wrote to his friend, Mensi von Klarbach, to thank him for sending Dessoir's article, announcing to write a reply correcting what he viewed as Dessoir's misleading statements: “That Schrenck is portrayed as the a[lpha] and o[mega] of Munich psychology leaves me cold, but Dessoir's characterization of the societies does not quite apply in the first place”.^29^ Du Prel's reply, also printed in the *Beilage zur Allgemeinen Zeitung*, started with a complaint regarding the irresponsibility of journalists presenting sensationalistic and misleading images of the work of the psychological societies. Regarding Dessoir's aversion to a transcendental psychology, du Prel reinstated his previous verdict according to which the study of hypnotism itself would eventually and inevitably result in a scientific need to consider a transcendental framework of the mind. He also reminded Dessoir that parts of his arguments, which were based on philosophical deductions from undisputed biological phenomena as well as from the psychology of dreams, aesthetics, and the philosophy of technics (e.g., du Prel, 1888), did not touch upon controversial areas ridden by public prejudice in the first place, implying that it was unfair of Dessoir to associate him with spiritism only.

To counter Dessoir's insinuation of du Prel's society being an ideological spiritist rather than a research association, du Prel remarked that it also involved skeptics, just as the GEP had members who were spiritists. Pointing to the inclusion of psychical research in the program of the first International Congress of Physiological Psychology in Paris, du Prel called to mind that the psychological societies abroad were composed of individuals with a diversity of convictions ranging from materialism to spiritism. Stressing the epistemic pluralism of the international psychological societies, du Prel stated that his association likewise admitted

doubters just as other psychological societies admit the spiritists. What we want is experiments; but where we differ from Herr Dessoir indeed is that we are perfectly indifferent as to where the experimentally observed natural facts will lead us, even if it was straight into metaphysics, nay, even into spiritism. We will adjust our minds according to the facts, not the other way around. ([1889] 1911, p. 227)

Du Prel then criticized Dessoir's *Doppel-Ich*. In his redefinition of the tasks of psychological societies, Dessoir had stated: “The much marveled-at automatic writing, inspired speech, crystal gazing—they all have now found their explanation in the fact of double-consciousness” (1889c, p. 2). Doubting the explanatory function of Dessoir's model, du Prel wrote: “The double-consciousness is merely the conceptual expression of the problem, but is in need of explanation itself. This is just the issue, to determine more precisely the bearer of this secondary consciousness of ours” (du Prel, [1889] 1911, p. 227). Charging Dessoir to willfully pass over facts incompatible with his scheme, du Prel claimed that a psychologist categorically excluding discussions of empirical material apt to clarify the nature of the soul, and the possibility of its survival of death, was like

a man milking the buck, while next to him stands a nanny goat with a bursting udder. This, however, Herr Dessoir does not do; he does milk the nanny goat, but he holds a sieve through which pass all those facts which he does not require for his aprioristic system. ([1889] 1911, p. 229)

The implicit charge of opportunism by the “spiritist” du Prel against the budding academic Dessoir gains an interesting momentum when we consider Dessoir's public coinage of the term “Parapsychologie” in the same year.^30^ Dessoir had written “Die Parapsychologie” (Dessoir, 1889b) in response to an article by a certain Ludwig Brunn in the March issue of the *Sphinx*, who, in accordance with Cesare Lombroso (a corresponding member of the GEP) and Adolf Bastian, maintained the intrinsically regressive and pathological nature of both genius and the belief in “mystical” or “supernormal” phenomena (Brunn, 1889). Without referring to the Brunn article, du Prel launched a direct attack on Lombroso in the June issue of *Psychische Studien*, arguing it was a fundamental error to confuse insanity as but one condition for the emergence of genius and “mystical” or supernormal experiences with their very cause (du Prel, 1889b).^31^ Dessoir published “Die Parapsychologie” as a reply to Brunn in the same month. Although much more cautious than du Prel, Dessoir made the same basic point and concluded that the “hitherto existing evidence for an exclusively pathological interpretation of parapsychology is insufficient” (Dessoir, 1889b, p. 344). Decidedly dumbfounding, however, is the result of our inquiry regarding Ludwig Brunn's identity. For, as revealed by Albert Moll (1893, p. 615n) and Alfred Fuchs (in his 1907 edition of Krafft-Ebing's *Psychopathia Sexualis*, p. 18), “Ludwig Brunn’ was none other than Max Dessoir himself.^32^

“Ludwig Brunn” had been one of several pseudonyms of Dessoir, who used the Brunn identity during a stint in sexology. Our state of befuddlement over the many faces of Dessoir approaches perfection, however, when we pursue his career further. In 1890, Dessoir made another, this time more lasting, turn by completely adopting the viewpoint of his alter ego, “Ludwig Brunn.” Written in 1890, Dessoir's article “Experimental pathopsychology” was published in the following year in the *Vierteljahrsschrift für wissenschaftliche Philosophie* (coedited by Wundt). Without offering an explanation for his radical change of mind, Dessoir now used the label “experimental pathopsychology” to cover the very class of phenomena he had previously defended from sweeping pathologization. Moreover, while in 1889 he still admitted an unexplainable rest of supernormal phenomena, he now plainly denied their existence, explaining, for example, that thought-transference could be reduced to muscle-reading (Dessoir, 1891, p. 93). Finally, and not less surprisingly, Dessoir now also expressed uncertainty regarding the very relevance of hypnotism and automatisms for scientific psychology (Dessoir, 1891, p. 62). While, as far as we know, this article was the only instance of Dessoir's explicit wholesale pathologization of the “supernormal” and statement of the nonexistence of telepathy, it would herald the beginning of a conservative and noncommitting stance toward the supernormal, which he was to maintain for the rest of his life (see, e.g., Dessoir, 1931, 1947b).

In 1889, both Dessoir and Schrenck-Notzing submitted papers to the International Congress of Physiological Psychology, which had been proposed by the Polish psychical researcher and psychologist Julian Ochorowicz and organized by members of the SPP, with Charles Richet serving as secretary.^33^ While Dessoir contributed a paper outlining his notions of the “double-consciousness” (Dessoir, 1890c), which was read in his absence, Schrenck gave two talks, one of which was an appeal to delegates to form an international association of psychology. Identifying the activities of the Berlin and Munich societies in Germany, the SPR in England, and the SPP in France, Schrenck advocated an interdisciplinary and eclectic research program for the proposed international association (Schrenck-Notzing, 1890a). Apart from his participation in the congress, 1889 was an overall important year for Schrenck to boost his young academic repute. For example, in January 1889, he gave a public lecture and demonstration of hypnotism before more than 300 mostly medical and scientific spectators at the Kunstgewerbehaus (house of art dealers) in Munich. After the talk, he demonstrated stages of hypnotism and induced a variety of hypnotic effects in eight different subjects, inviting physicians in the audience to test the hypnotic trance on the spot. He also highlighted the aesthetical dimensions of hypnotism by inducing refined emotional expressions in the hypnotic trance of Baron von Poyßl, a member of the MPG (Hübbe-Schleiden, 1889a).^34^ In October of the same year, Schrenck gave another talk on the practical implications of hypnotism before a large audience at the *Kaufmännische Verein* (Society of Merchants) in Munich (Schrenck-Notzing, 1889).

Considering Schrenck's appeal for international collaboration at the International Congress of Psychology, it seems likely that the impetus for the fusion of the MPG and GEP in 1890 came from Schrenck rather than from Dessoir. At any rate, there was no visible collaboration between Schrenck and Dessoir worth mentioning prior to the schism in the MPG, and there is reason to believe that the young men considered each other as competitors in the introduction of medical hypnotism in Germany, which is reflected in their apparent race for authority through their many contributions on hypnotism to the *Sphinx*. That jealousy appeared to have been an issue was reflected in Schrenck's writing a critical review of Dessoir's acclaimed bibliography of hypnotism (Dessoir, 1888a) in the *Sphinx*, where young Schrenck scolded his colleague for the omission of what he deemed major references ([Schrenck-]Notzing, 1888a). Dessoir swiftly reciprocated with a rather snappish review of Schrenck's published MD thesis in sexology (Schrenck-Notzing, 1888) in *Psychische Studien* (Dessoir, 1888c).

Prior to this open conflict, it seems Schrenck had invited Dessoir in 1887, when the MPG was still dominated by du Prel's agenda, to become a honorary member (außerordentliches Mitglied) of the Munich society, which, in a letter to Hübbe in August 1887, Dessoir refused.^35^ A few weeks later, Dessoir asked Hübbe rather than Schrenck if it was possible to inspect the records of some experiments conducted by the latter, of whose results Hübbe had apparently informed him.^36^ The only known request of a mutual collaboration with the MPG by Dessoir prior to the exodus of the du Prel wing was in November 1887, when Dessoir asked Hübbe whether the MPG was interested in conducting joint mediumistic experiments in France.^37^ Shortly after the foundation of the GEP in 1888, du Prel rather than Schrenck seemed to have contacted Dessoir to obtain information about planned activities (and possibly to suggest collaboration, though this is not clear from Dessoir's letter). Dessoir wrote to Hübbe and asked him to convey a message to the MPG stating that the GEP first needed to sufficiently consolidate and present positive results to the public before an expansion could be considered.^38^ However, a few months later, Dessoir expressed hope for a fruitful collaboration with Munich despite, he admitted, certain divergences in perspective. Apparently having forgotten his previous rejection of the invitation to join the MPG, Dessoir wrote to Hübbe: “To start with a good example I ask your permission to join your Society, which I haven't done to date because of the very simple reason of my nonage, which terminated only recently.”^39^

This was shortly before Schrenck's critique of Dessoir's bibliography, and it seems that it was only after the schism between Schrenck and du Prel in 1889 that Dessoir and Schrenck buried their differences, started communicating, and even promoted one another's work. For example, Dessoir would describe the acceptance of Schrenck's thesis on the therapeutic effects of hypnotism by the prestigious medical faculty of Munich university as an important breakthrough for medical hypnotism in Germany, comparing its import with that of the installment of the first regular lecture series at a German university on hypnotism by Wilhelm Preyer in Berlin in the following year (Dessoir, 1889a).

Although Schrenck's professed change of perspective was less radical than that of Dessoir, his rejection of the former mentor were just as active and programmatic. The first public attack on du Prel occurred in 1889, when Schrenck reviewed an essay by du Prel regarding the problem of hypnotic crime (du Prel, 1889a). Using the pseudonym “Franz Imkoff,”^40^ Schrenck found several factual errors, criticized du Prel for strongly exaggerating the possibility of a perfect crime through hypnosis, and, like Dessoir and Hartmann, rejected du Prel's transcendental-psychological deductions from the literature of animal magnetism. Moreover, Schrenck, his own published experiments and statements to the contrary notwithstanding, now also held that not even recent empirical evidence for telepathy and clairvoyance was yet conclusive, criticizing du Prel for drawing momentous consequences such as the reality of postmortem survival from distinctively insufficient data (Imkoff, 1889).

## The Fusion of the GEP and MPG and its Aftermath

The fusion of the GEP and MPG into the “Gesellschaft für psychologische Forschung” (GfpF) in November 1890 marked a point of no return, particularly in Dessoir's agenda to normalize controversial phenomena related to hypnotism. In addition to the overwhelming hostility of orthodox science, the church, the law, and not least the liberal German press to spiritism and occultism and anything that appeared remotely associated with it,^41^ representatives both of fledgling academic psychology and of medical hypnotism felt the need to radically sever any links between their fledgling disciplines and the “experimental metaphysics” of spiritism and animal magnetism, of which du Prel was the most popular proponent in Germany. The “disenchantment” of hypnotism was on the agenda of figures much more established than Dessoir and Schrenck, such as Wilhelm Preyer (1878), August Forel (1891, 1894), Oscar Vogt (1898, 1901), and, most militantly, Albert Moll (1892, 1903), who became one of the most active and influential members of the GEP and GfpF. Hence, young men such as Dessoir and Schrenck, who actively supported an expansion of nascent German psychology at critical junctions of their academic careers as pioneers of hypnotism, realized that it was hopeless to continue taking aboard the study of telepathy and other “occult” phenomena associated with hypnotism.

The last *Sphinx* article signed by Schrenck with his actual name was thus published in 1889 and concerned hypnotherapy rather than psychical research ([Schrenck-]Notzing, 1889). While Dessoir published his last *Sphinx* paper under his real name in the year of the fusion of the GEP and MPG (Dessoir, 1890a), Schrenck continued to use the Imkoff pseudonym for *Sphinx* articles that doubted the existence of supernormal phenomena and that were concerned with the medical and legal implications of hypnotism only (e.g., Imkoff, 1890, 1891a, 1891b). In 1891, Schrenck also ceased to contribute to the *Sphinx*, which continued to publish papers by du Prel until 1893.

Around the time of the fusion of the GEP and MPG, Dessoir was preparing a career in physiological psychology, working on his MD degree in Hermann Munk's laboratory in Berlin. William James’ student Charles Augustus Strong visited Munk's laboratory in January 1890, where he also met Dessoir, with whom he experimented on frogs. Strong reported to James that he attended a meeting at the GEP and that

at Dessoir's suggestion [I] think I will apply for admission to it. A singularly prepossessing thoughtful, well-spoken set of laymen are its members. Dessoir read a brilliant paper on the different disciplines of psychology and the relations of each to hypnotism; I am to hear him finish it to-morrow night. (Charles Augustus Strong to William James, January 5, 1890, in Skrupskelis & Berkeley, 1999–2004, vol. 7, pp. 2–3)^42^

According to Max Offner (1891), a psychologist specializing in the problems of memory and fatigue who served as vice-secretary of the Munich section of the GfpF, honorary members of the society included Liébeault, Bernheim, Jules Liégeois, Richet, Sidgwick, Myers, Lombroso, William James, Joseph Jastrow, von Hartmann, and others.^43^ Prior to the fusion, the GEP honorary membership also seemed to have included, apart from Lombroso and Bastian, Eugen Bleuler (Bentivegni, 1890, p. 33).^44^ One of the greatest triumphs, however, was the recruiting of Hugo Münsterberg, whose public attack on the MPG and GEP in 1889, we recall, contributed to Dessoir's adapting a decidedly conservative stance to the study of supernormal phenomena. In telling contrast to his previous denouncements not too long ago, Münsterberg now even published a long essay in the *Schriften* (transactions) of the GfpF, fittingly titled “On the tasks and methods of psychology” (Münsterberg, 1891). Moreover, Dessoir and Münsterberg quickly became close friends, and Münsterberg biographer Matthew Hale (1980, p. 113) refers to Schrenck as a collaborator of Münsterberg.^45^

Max Offner's article outlined the work and objectives of the newly founded GfpF, revealing that the program was nearly identical with the revised GEP program publicized by Dessoir in 1889. Like Dessoir before him, pointing to du Prel and his group, Offner excused the previous rejection of psychical research by German representatives of academic psychology purely in terms of methodological shortcomings, lack of critical thinking, and the “need to believe” displayed by some of its proponents. Like Dessoir, Offner identified the split between du Prel's camp and the MPG as a decisive step toward the needed approximation of psychical research to official science, claiming: “The Psychological Society, however, stands on the positive ground of normal psychology and seeks, in close proximity to official science, to extend the inductive method to anomalous psychological phenomena” (Offner, 1891, p. 334 n331). Using another pseudonym to write in the *Sphinx* (Rells, 1891), Dessoir seconded Offner's portrayal of the Munich branch and lauded Schrenck-Notzing's renunciation of his own previous positive experiments in telepathy (partly conducted with du Prel), which had just been republished in du Prel's latest book (du Prel, 1890, vol. 2).

However, although Schrenck did made a remark to this effect (Schrenck-Notzing, 1891b, p. 14 n1), it is vital to note that this was in a preface to his translation of Charles Richet's studies in telepathy and clairvoyance (including a response to Preyer), which reported positive effects, and that the rest of Schrenck's preface painted a cautious but fairly optimistic picture regarding the scientific status of certain supernormal phenomena. Also, the same year saw Schrenck's publication of an apparently successful telepathy study in the *Proceedings* of the SPR (Schrenck-Notzing, 1891a). Hence, although Schrenck certainly adopted a markedly conservative stance at least in his German publications, Dessoir's characterization of Schrenck's new position was perhaps more an appeal to his colleague in Munich rather than a truthful epistemological stock check. Moreover, not all members of the Berlin section of the GfpF seemed to be in line with Dessoir. For example, in 1891, the *Sphinx* published the German translation of Charles Richet's preface to the French edition of a classic SPR study on “telepathic hallucinations,” which was circulated separately by the GEP (Richet, 1891). Also, the translation by the MPG of a (positive) paper on clairvoyance by Ambroise-Auguste Liébeault (1891) in the same year suggests that the new orientation of the Munich group was not as uniform as Dessoir and Offner tried to imply.

The ambivalent orientation of the GfpF is also confirmed by Henry Sidgwick's impressions during his visit to Berlin and Munich in the following year. An entry in Sidgwick's diary on April 7, 1892, tells of his visit in Berlin, where he met Wilhelm Preyer and Hermann Ebbinghaus, as well as members of the Berlin branch of the GfpF:

I went to a meeting of the “Psychologische Gesellschaft”, which corresponds to the S.P.R. I was received with marked politeness, and liked the members; but the society does not appear to succeed in doing any “psychical research”, and has to get matter for its meeting by digressing into orthodox psychology. (E. M. Sidgwick & Sidgwick, 1906, pp. 516–517)

In a letter from Munich to his wife Eleanor a few days later, Sidgwick reported that upon his arrival in Munich, he was greeted by Max Offner

who rather represents the side of orthodox psychology, and is not convinced of telepathy—but not hostile. The Gesellschaft in its present form combines ordinary psychology with psychical research: and doesn't do much at the latter from want of subjects:^46^ in fact, I gather that what they chiefly do in this line is from time to time to have a report on our *Proceedings*!” (H. Sidgwick to E. M. Sidgwick, April 15, 1892, in E. M. Sidgwick & Sidgwick, 1906, pp. 519–520)

However, “relapses” by GfpF members into psychical research notwithstanding it is obvious that the *Schriften* of the GfpF actively catered to a decidedly orthodox audience. As we remember, the first installment contained a paper by former MPG and GEP critic Hugo Münsterberg, and other contributions were no less conventional in scope, involving papers by further psychological luminaries such as Theodor Lipps (1897, 1902), Arthur Wreschner (1898), William Stern (1900) and Paul Möller (1905). In fact, the *Schriften* served as a forum to actively repudiate unwanted associations between “orthodox” hypnotism and psychology with the supernormal, such as August Forel's forensic report on a case of pseudo-clairvoyant somnambulism (Forel, 1891), Albert Moll's study of the hypnotic rapport (Moll, 1892), and Edmund Parish's critique of the “Census of hallucinations” conducted by the SPR in collaboration with the International Congress of Psychology (Parish, 1894).

Schrenck, however, was still active in psychical research, continuing his “enlightenment crusade” to cleanse it both from spiritualism in general and from du Prel's influence in particular, using formal and informal opportunities to scientifically disqualify du Prel in the still considerably overlapping international communities of psychology, hypnotism, and psychical research. For instance, in a letter to his friend Mensi von Klarbach, du Prel had written in 1889: “Our new society did not have any delegates at the hypnotic congress in Paris; Schrenk is said to have blackened us there greatly.”^47^ In 1892, it seems it was because of Schrenck's influence that Alexandr Aksakov's suggestion to Frederic Myers to propose du Prel for election as an honorary or corresponding member or associate of the SPR was turned down. In his reply to Aksakov, Myers expressed concerns about the philosopher's scientific standing. After all, du Prel had

never done any real work in investigation, & has merely *hashed up old stories* without even trying to repeat experiments, etc. Baron von Schrenck-Notzing,—a most capital & delightful fellow, whom we all trust thoroughly,—did not find du Prel worth much as a scientific coadjutor.^48^

While Dessoir and Albert Moll worked on the public repudiation of occult remnants in hypnotism, Schrenck tackled a related issue, namely, du Prel's recent embracing of Reichenbach's concept of the so-called “odic force,” which bore close resemblance to the “magnetic fluid” of mesmerism. Karl Ludwig von Reichenbach (1788–1869), the discoverer of paraffin and creosote, claimed to have discovered a subtle force that was supposed to be visible to specially predisposed persons or “sensitives.” After the SPR had failed to replicate Reichenbach's findings and concluded that most effects reported by his “sensitives” were likely due to unwitting suggestion by the experimenter, the scientific cash value of the “odic force” diminished considerably in psychical research.^49^ Still, du Prel, who did not read English and was generally not very interested in the SPR work, would ignore the argument from suggestion and, from the early 1890s onward, increasingly framed his own ideas in terms of Reichenbach's “odic force” (see, e.g., du Prel, 1890, 1894, 1899). In 1891, Schrenck reissued one of Reichenbach's studies to show, through a preface and numerous annotations, that the effects reported by Reichenbach were due to nothing but suggestion (Schrenck-Notzing, 1891c).^50^

The year 1892 saw further events that proved significant for the careers of Dessoir and Schrenck. Dessoir continued his path into the safe havens of academia by obtaining his second doctorate, that is, an MD degree with a thesis on the “Hautsinn” (“skin sense”), as well as his habilitation (the German formal precondition to obtain a professorship).^51^ Schrenck, who by now had earned international repute as an authority in both hypnotism and sexology *en par* with authors such as August Forel, Richard von Krafft-Ebing, Havelock Ellis, and Albert Moll, published a highly acclaimed book on the hypnotherapeutic “treatment” of homosexuality (Schrenck-Notzing, 1892) and continued his successful practice as a physician and hypnotherapist. Along with Moll, Sigmund Freud, and other renowned authors, both Schrenck and Dessoir joined the editorial board of the newly founded *Zeitschrift für Hypnotismus* (*Journal of Hypnotism*), which coincided with Hübbe-Schleiden's decision to change the focus of the *Sphinx* from psychical research to theosophy. This prevented Dessoir and Schrenck from continuing to associate their names (and even pseudonyms) with the *Sphinx*, and by 1893, du Prel also ceased to supply the journal with articles.

Also in 1892, Wilhelm Wundt launched another strike against psychical research in an essay on hypnotism published in his *Philosophische Studien* and separately as well as in the second edition of his *Lectures on Human and Animal Psychology*. Both texts contained direct attacks on the psychological societies in Munich and Berlin (Wundt, 1892, passim; 1894, pp. 335–336). After Münsterberg had been won over by Dessoir and Schrenck and would now attack du Prel only (Münsterberg, 1892, p. 113), the gist of the argument presented by the father of German academic psychology was that scientific psychologists ought to steer clear from hypnotism because of its associations with the “occult.”^52^ Despite visible and apparently radical changes in their research foci and epistemological orientation, Wundt expressed serious concerns about the work of the psychological societies in Berlin and Munich and of their equivalents in England and France. Wundt was particularly concerned about the active involvement of such societies in the making of scientific psychology as reflected in the first International Congress in 1889 and the impending second Congress in London, which was organized by the SPR (Henry Sidgwick served as president, and, together with James Sully, Frederic Myers as secretary). As in his 1879 pamphlet, Wundt warned that empirical research of supernormal phenomena undermined cultural progress by offering inroads for superstition and magical thinking, which Wundt continued to view as fundamental threats for the social, religious, and moral order and modern civilization.^53^ Dessoir, who would later write in his memoirs that following his 1889 article on the scope and methods of the psychological societies both Wundt and Oswald Külpe had “raised their warning voice” (Dessoir, 1947a, p. 127), reviewed Wundt's hypnotism paper in the *Zeitschrift für Hypnotismus*. Expressing his complete agreement with Wundt's concerns about the disastrous societal consequences of superstition, Dessoir begged to differ regarding Wundt's portrayal of modern hypnotism research as conducted by the GfpF (Dessoir, 1892). Schrenck, however, seemed anxious to avoid any associations with psychical research at the London Congress, in which he was visible only as a discussant of a paper by Edgar Bérillon on hypnotism as a pedagogic tool (Bérillon, 1892) whereas Dessoir again did not seem to have attended.

Yet, 1892 was the year that ultimately decided Schrenck's future as the doyen of early twentieth-century German psychical research. By marrying Gabriele Siegle, daughter and heir of the Swabian industrialist Gustav Siegle, in 1892, Schrenck became one of the wealthiest men in Munich. His new economic independence allowed him to travel widely to investigate, mostly in collaboration with Charles Richet and Julian Ochorowicz, mediums such as the notorious Eusapia Palladino, who had just converted the former arch-skeptic Cesare Lombroso. However, it would take almost a decade before Schrenck would publish results of his mediumistic investigations, and up to du Prel's death in 1899, his battle against spiritualism in general and his former mentor in particular would continue unabated.

All the while, Schrenck fostered his career in hypnotherapy, sexology,^54^ and psychology. Although there is no indication that Carl Stumpf in Munich was among the members of the GfpF, there were links to Schrenck-Notzing, who hosted Stumpf's friend William James in February and March 1893.^55^ In his first letter from Munich to his wife Alice, James referred to Schrenck as a “psychical researcher” rather than a psychologist or physician (Skrupskelis & Berkeley, 1992, vol. 7, p. 384). James mentioned another dinner, this time at Schrenck's, with Stumpf and his wife as guests, after which Schrenck tried his method of hypnosis on the American guest of honor.^56^

In 1895, Schrenck published another critically acclaimed sexological study (Schrenck-Notzing, 1895a) and became, together with Wilhelm Preyer and others, a legal expert in an alleged case of hypnotic crime that caused a great stir in the German press (Grashey et al., 1895). With Carl Stumpf as president, he also started coordinating the third International Congress of Psychology to be held in Munich in the following year. Possibly in anticipation of the Congress in his home country and of the continued involvement of psychical researchers in its organization, Wilhelm Wundt made a third attempt to demarcate psychology from psychical research by publishing a study by the Danish psychologists Hansen and Lehmann (1895) in his *Philosophische Studien*, which aimed to demonstrate that telepathy experiments not explainable in terms of Preyer's “muscle-reading” hypothesis were in fact due to the sender's transmission of information by “unconscious whispering.” Although Henry Sidgwick (1896, 1897) would later refute the main points of the study, Schrenck, apparently careful not to take a controversial stance before acting as the third International Congress’ secretary, published a review of the Hansen and Lehmann paper completely embracing the authors’ skeptical results and conclusions (Schrenck-Notzing, 1895b).

However, despite Schrenck's demonstrated caution, his continued interest and involvement in psychical research still appeared to meet other psychologists’ reservations. For instance, Henry Sidgwick, upon returning from the Munich Congress, wrote to William James on August 21, 1896, that Schrenck's position as general secretary was only “allowed with some hesitation” (Skrupskelis & Berkeley, 2000, vol. 8, p. 185). Sidgwick's claim is consistent with a remark by Carl Stumpf, who would later reminisce: “In 1896 von Shrenck-Notzing [sic] and I took charge of the preparations for the Third International Congress of Psychology in Munich, also of its direction […]. I endeavored to prevent hypnotic and occult phenomena from occupying the foreground, as had been the case in former sessions” (Stumpf, 1930, p. 404).

## Conclusion

While Schrenck ultimately took his departure from sexology and hypnotherapy research by 1911 to devote himself almost exclusively to the investigation of mediumship and poltergeist phenomena for the rest of his life, the *Schriften* of the GfpF continued to be published until 1916. In 1906, Schrenck appeared to still support the society financially. This is implied by a letter from Hübbe-Schleiden to fellow theosophist Ludwig Deinhard in November 1906, where Hübbe expressed astonishment about a recent resolution in the GfpF, according to which foreign members were to be reimbursed for their travel expenses, which, Hübbe remarked, was a considerable sum in the case of Hugo Münsterberg, who meanwhile was running the psychology laboratory at Harvard.^57^

The Berlin section became increasingly dominated by Albert Moll, who used the society as a forum for his ruthless war against proponents of both psychical research and alternative and lay medicine (Kurzweg, 1976; Sommer, 2012b). When Schrenck published his controversial first study on the phenomena of materializations shortly before the outbreak of World War II (Schrenck-Notzing, 1914), both Moll and Dessoir positioned themselves—in the name of “science,” “rationality’, “civilization,” as well as “true religion”^58^—as vocal public opponents. Despite support from scholars of the caliber of Charles Richet, who had won the Nobel Prize in physiology for his work in anaphylaxis in 1913, the biologist Hans Driesch and Swiss psychiatrist Eugen Bleuler, Schrenck and other German psychical researchers were easy prey to polarizing defamations and deadly ridicule in the press, which self-styled guardians of science such as Moll, the Potsdam court district director Albert Hellwig, and Max Dessoir had learnt to utilize (Wolffram, 2006; Sommer, 2012b).^59^ It seemed, in fact, that not much had changed since Resau.

A close analysis of the formation of the GfpF and the early careers of its founders, which intersected with the making of modern psychology at a critical junction, reveals that the German example is also apt to revise popular presentist historiographies of psychical research framing its demise in simplistic terms of the victory of science and rationality over stubborn remnants of superstition (e.g., Hall, 1962; Alcock, 1981; Brandon, 1983). Contrary to the founders of the SPR or William James, neither Schrenck nor Dessoir, who, we remember, were both young men yet to complete their degrees when embarking on the expansion of nascent German psychology, possessed the social status, academic reputation, experience, money, and influence that the project of integrating psychical research into the still moldable science of psychology would have required if it was to stand a chance in the first place. The later career of Schrenck, whose true intellectual passion would fully develop only after gaining complete economical independence, also confirms this view. Similarly, although du Prel, who had buried his hope of an academic career as early as 1875 (Kaiser, 2008, p. 43), did not enjoy financial independence, the private scholar had nothing to lose in terms of academic repute and therefore nothing to retract either.^60^

Finally, far from strictly limiting itself to a national context, the German example offers a novel analytical angle for an appreciation of the fundamentally opposed visions and agenda of the very “fathers” of modern psychology, Wilhelm Wundt and William James. While Wundt remained opposed to psychical research and hypnotism, James became increasingly influenced by the ideas of Frederic W. H. Myers (see, e.g., Taylor, 1983, 1996; Kelly et al., 2007), whose theory of the “subliminal self” bore close resemblance to du Prel's “transcendental subject” (Sommer, 2009). James, who was familiar with du Prel's writings and who visited the philosopher during his stay in Munich in 1893 to invite him to conduct mediumistic experiments in Italy,^61^ also criticized du Prel for his lack of scientific rigor, expressing preference for the very similar theoretical framework that Myers developed on more rigorous empirical grounds (James, 1894). Attacks by representatives of academic psychology and psychical research alike notwithstanding, du Prel was the most important popular voice in Germany expressing a concern shared by men such as Sidgwick, Myers, and James: the promotion of empirical studies of alleged transcendental functions of the psyche as a potential Gordian knot to scientifically refute materialism, at the same time offering a rational, evidence-based foundation for a progressive and antidogmatic religious reform. Hence, when viewed on the backdrop of parallel international historical developments, the history of the GfpF throws into sharp relief fundamental concerns regarding the legitimate scope of modern psychology as debated during its infancy.

## Footnotes

1 On Schrenck's pioneering work in hypnotism and sexology, see Ellenberger (1970), Gauld (1992), and Sulloway (1992). Regarding the controversies around his studies of physical mediumship, see, for example, Wolffram (2009) and Sommer (2012b).

2 For contributions of the SPR to early psychology, see, for example, Alvarado (2002), Crabtree (1993, 2003), Ellenberger (1970), Gauld (1992), Shamdasani (1993, 1994), Sommer (2011) and Taylor (1983, 1996). On the founders of the SPR, see Gauld (1968), Oppenheim (1985), Williams (1984), and Hamilton (2009).

3 Bringmann, Bringmann, and Ungerer (1980, p. 146) found that Wilhelm Wundt originally intended to name his journal *Psychologische Studien* but changed the title to *Philosophische Studien* because, the authors suggest, Wundt was concerned that readers might associate his journal with Aksakov's periodical (however, Bringmann et al. erroneously refer to the latter as *Psychologische* rather than *Psychische Studien*).

4 On July 22, 1885, du Prel wrote to his friend, the famous painter and future member of the MPG, Gabriel Max: “The foundation of a spir.[itistic] monthly is being discussed, which shall be supplied mainly by [Hellenbach], Schleiden, Hartmann and me, but which is also supposed to cover experimental psychology, theosophy, etc.” (Max papers, Münchner Stadtbibliothek, Monacensia GNM I, C-201). Du Prel used terms such as “spiritism,” “mysticism,” “magic,” etc., in a highly ambivalent manner, often as equivalents of “psychical research.” (Unless specified otherwise, translations from the German are always mine.)

5 Although du Prel implied his “monistic soul doctrine” to be an ontological one, it can pass as a psychological monism at best.

6 According to Hübbe's notebook of 1884–1885, another subtitle under consideration had been “Scientific monthly for experimental psychology with consideration of the ‘mystical’ and ‘magical’ explanations of the orient and the occident” (Hübbe-Schleiden papers, Niedersächsische Staats- und Universitätsbibliothek Göttingen, Cod. Ms. W. Hübbe-Schleiden 1012/4).

7 Du Prel published most of the articles later constituting *Die monistische Seelenlehre* in the *Sphinx*.

8 An English version of this article was published in *Science* (Dessoir, 1887c).

9 Although *A Century of Serial Publications in Psychology, 1850–1959* (Osier & Wozniak, 1984) lists most leading nineteenth-century psychical research periodicals such as *Psychische Studien* in Germany, the *Proceedings* of both the English and American SPR and the *Annales des sciences psychiques* in France, the *Sphinx* does not seem to have been included.

10 However, Aksakov's *Psychische Studien*, the competitor of *Sphinx*, occasionally published communications of the MPG as well. See, for example, [Schrenck-]Notzing (1888c).

11 Although the program was published anonymously, the identity of the author with du Prel is verified by the inclusion of the text in his posthumous writings (du Prel, 1911), as well as by a letter to Hübbe on October 28, 1886, in which du Prel announced to expand §1 of the constitution into a full program for publication in the *Sphinx* (Cod. Ms. W. Hübbe-Schleiden 79/8, see also Kaiser, 2008, p. 88n343).

12 The Hübbe collection holds letters from Dessoir between 1886 and 1888 and one letter from 1890.

13 Dessoir to Hübbe-Schleiden, no date (probably July 20, 1886), Cod. Ms. W. Hübbe-Schleiden 68/3.

14 Like Hübbe, Hartmann is also mentioned but briefly in Dessoir's memoirs. Dessoir wrote that he owed much to Hartmann in his “twenties and thirties,” acknowledging Hartmann's advice regarding career moves (Dessoir, 1947a, p. 33). However, Dessoir's frequent references to Hartmann in his correspondence with Hübbe confirms Kurzweg's (1976) suggestion that Hartmann's influence had started before Dessoir turned 18. Kurzweg also argues that von Hartmann's influence on the research program of the GEP was considerable (pp. 125–128, 319).

15 Dessoir to Hübbe-Schleiden, August 1, 1887 (Cod. Ms. W. Hübbe-Schleiden 68/36).

16 Dessoir to Hübbe-Schleiden, August 13, 1887 (Cod. Ms. W. Hübbe-Schleiden 68/37, original emphases).

17 Hübbe made efforts to initiate a constructive discourse with proponents of orthodox psychology. For example, he sent complimentary copies of the *Sphinx* to several psychologists and medical hypnotists, such as Rudolf Heidenhain, who briefly acknowledged receipt on January 30, 1886 (Cod MS W. Hübbe-Schleiden 331/217). Hübbe also invited Wilhelm Preyer to contribute an article concerning the SPR to the *Sphinx*. Preyer replied, also on January 30, 1886, to send his thanks for the copy of *Sphinx* but declined the invitation by referring Hübbe to his *Deutsche Rundschau* article (Cod. Ms. W. Hübbe-Schleiden 231).

18 The process of the GEP's formation on January 31, 1888, is outlined in Dessoir's letters to Hübbe-Schleiden on January 9, 13, and 22, 1888, and February 1, 1888 (Cod. Ms. W. Hübbe-Schleiden 68/44–47), where Dessoir briefly reported on two preconstitution meetings, in which the adoption of the SPR research program was agreed upon, and that the society was to go public in late 1888 only. On January 22, 1888, Dessoir wrote to Hübbe that despite his “most severe resistance” (“trotz heftigsten Strauebens”), he was elected the society's secretary (Cod. Ms. W. Hübbe-Schleiden 68/46).

19 For instance, on June 26, 1889, du Prel wrote to his friend and biographer, Alfred Mensi von Klarbach, that “Schrenk is to be blamed for the whole affair” (Mensi von Klarbach papers, Bayerische Staatsbibliothek München, 15/74). In his letters, du Prel usually referred to Schrenck-Notzing as “Schrenk” (a typographic precursor of “Schrenck”).

20 In 1884, the adolescent Dessoir had reproduced the “muscle-reading” tricks of stage magician Stuart Cumberland. Dessoir's feats received some press coverage; see Kurzweg (1976, p. 97).

21 Minutes of Council Meetings. October 2, 1885, to November 29, 1889. Vol. III. July 22, 1887, M2. SPR archive, Cambridge University Library. Dessoir expressed his pride about his election to Hübbe; see Cod. Ms. W. Hübbe-Schleiden 68/29 & 40.

22 As a child, Dessoir had gained some fame as a violin prodigy, while Gurney, prior to his commitment to psychical research, was an accomplished music theorist. On Gurney's contributions to psychology, see Gauld (1992) and Sommer (2011).

23 Moll was at the forefront of German physicians struggling to establish hypnotism as a legitimate field of medicine by demonstrating its therapeutic effects, on the one hand, and by aggressively cleansing it from its “mystical” connotations, on the other hand (Kurzweg, 1976; Sommer, 2012b).

24 Cod. Ms. W. Hübbe-Schleiden 68/46.

25 Dessoir to Hübbe, February 9, 1888 (Cod. Ms. W. Hübbe-Schleiden 68/48).

26 Dessoir to Hübbe, April 10, 1888 (Cod. Ms. W. Hübbe-Schleiden 68/51).

27 Dessoir to Hübbe, July 7, 1887 (Cod. Ms. W. Hübbe-Schleiden 68/30). Hübbe did not accept Dessoir's proposal.

28 *Das Doppel-Ich* was published in 1889 despite its official publication date (1890).

29 Du Prel to Mensi von Klarbach, October 25, 1889, Klarbach papers, 20/74.

30 Although proposed in print by Dessoir as early as 1889, the term became widely adopted in Germany in the late 1920s only and introduced into English in the early 1930s by the founder of experimental parapsychology J. B. Rhine (see, e.g., Mauskopf & McVaugh, 1980).

31 At the same time, du Prel attempted to rebut von Hartmann's previous claim regarding the pathological nature of somnambulism by citing anecdotes from the Romantic medical literature reporting instances where induced somnambulism did not appear to cause but rather cure insanity.

32 As far as I'm aware, the first to unveil the identity of Max Dessoir and “Ludwig Brunn” in recent times was Gerd Hövelmann, to whom I'm indebted for sharing his discovery. See also Hövelmann (forthcoming). Another pseudonym used by Dessoir was “Edmund W. Rells.”

33 Hübbe (1889b) published a personal invitation by Richet to *Sphinx* readers to participate in the congress. On the involvement of psychical researchers in this first International Congress of Psychology, see, for example, Brower (2010) and Plas (2000).

34 Schrenck's second paper at the International Congress of Psychology in Paris also concerned the aesthetic implications of hypnotism, involving a presentation of photographs of dramatic emotional expressions in Schrenck's hypnotic subjects (Schrenck-Notzing, 1890b). In 1904, he would organize another public performance involving “hypnotic art,” which earned him the wrath of Albert Moll: the performances of the French “dream dancer” Magdeleine G. (Schrenck-Notzing, 1904). On the resulting conflict with Moll, see Wolffram (2009) and Sommer (2012b).

35 Dessoir to Hübbe, August 31(?), 1887 (Cod. Ms. W. Hübbe-Schleiden 68/40).

36 Dessoir to Hübbe, September 3, 1887 (Cod. Ms. W. Hübbe-Schleiden 68/41).

37 Dessoir to Hübbe, November 23, 1887 (Cod. Ms. W. Hübbe-Schleiden 68/42).

38 Dessoir to Hübbe, February 9, 1888 (Cod. Ms. W. Hübbe-Schleiden 68/48).

39 Dessoir to Hübbe, March 5, 1888 (Cod. Ms. W. Hübbe-Schleiden 68/50).

40 The identity of Schrenck with “Franz Imkoff” was revealed by his secretary and biographer, the philosopher Gerda Walther (1962).

41 On this point, see, for example, Freytag (2004) and Treitel (2004).

42 However, shortly afterward Strong apparently revised his favorable opinion: “I fear that Dessoir […] with all his learning, fecundity, & skill in exposition, is but a mediocre reasoner” (Strong to James, May 13, 1890, Skrupskelis & Berkeley, 1999–2004, vol. 7, p. 25).

43 According to a press release in 1892, the membership of the GfpF comprised 56 regular, 109 honorary, and 12 corresponding members (“Die Organisation der Gesellschaft für psychologische Forschung,”).

44 Incidentally, both Lombroso and Bleuler later became convinced of the reality of telepathy and even the alleged physical phenomena of mediumship. See, for example, Lombroso (1909) and Bleuler (1929, 1930).

45 Dessoir writes about his friendship with Münsterberg in his memoirs (Dessoir, 1947a, pp. 162–164).

46 The lack of suitable subjects for research appeared to have been a general problem for German psychical research even before the fusion. For example, in his letter to Hübbe on January 22, 1888, Dessoir complained that want of subjects severely delayed the consolidation of the newly founded GEP (Cod. Ms. W. Hübbe-Schleiden 68/46).

47 Du Prel to Klarbach, November 6, 1889 (Mensi papers, 19/74).

48 Frederic W. H. Myers to A. Aksakov, May 10, 1892, original emphasis (Aksakov papers, Pushkinskij Dom, St. Petersburg, not indexed). Schrenck had been elected a corresponding member of the SPR in April 1888.

49 For a history of Reichenbach's “odic force,” see Nahm (forthcoming).

50 Schrenck's move was reminiscent of the English “father of hypnotism,” James Braid (1795–1860), who had also argued that Reichenbach's results were due to suggestion (Braid, 1846).

51 The topic of his disputation was “On the belief of the mentally ill in the reality of hallucinations” (Zwikirsch, 1994). Unfortunately, I was unable to establish whether a published version of the talk exists. Obviously, it would be interesting to ascertain to what extent the talk corresponded with views expressed in Dessoir's (1891) article “Experimentelle Pathopsychologie.”

52 While Wundt and other psychologists in Germany either ignored or attacked the Berlin and Munich psychological societies, foreign psychologists were more generous. For example, from its inception in 1887, even the *American Journal of Psychology* (*AJP*, founded and edited by G. Stanley Hall, one of the most ruthless opponents of William James’ attempts to integrate psychical research into American psychology) had summarized papers appearing in the *Sphinx*, and it published a note on the foundation of the GEP (“Notes,” 1888, pp. 735–736). In an overview of psychological work conducted in German cities, University of Illinois psychologist William O. Krohn (1892) also acknowledged the work of the GfpF. Regarding Munich, Krohn wrote in the *AJP* that he was “indebted to Dr. Freiherr von Schrenck-Notzing” for showing him “much in the way of hypnotic experiments. This well-known physician constantly makes use of hypnotism as a therapeutic agent. He is also a close student of psychology, as Stumpf, Edward [sic] von Hartmann and others testify” (p. 589). Krohn also mentioned the work of Dessoir's Berlin section and both groups’ involvement in psychical research (p. 590).

53 In his presidential address to the Congress, Sidgwick referred to Wundt's charges and chided him for expressing “opinions on matters on which he is determined to seek no information” (Sidgwick, 1892, p. 2), anticipating a similar protest presented by Frederic Myers in the pages of *Mind* (Myers, 1893).

54 See, for instance, his talk at the Anthropological Society in Munich in 1893 (Schrenck-Notzing, 1893).

55 James and Schrenck seemed to have met at the first International Congress of Psychology in Paris. In a letter to Carl Stumpf on August 15, 1889, James had lamented that “there were only 2 germans [sic], Münsterberg, and one Schrenk-Notzing [sic] from Munich” (Skrupskelis & Berkeley, 1998, vol. 6, p. 526).

56 Although Schrenck was able to produce “only an incipient effect” in James (James to A. G. J., probably February 28, 1893, Skrupskelis & Berkeley, vol. 7, 1999, p. 386), he seemed sufficiently impressed to recommend the Bavarian as a therapist. For example, in March 1893, James wrote to Violet Paget, who was looking for help for her half brother Eugene Lee-Hamilton: “von Schrenk in Munich, who is personally an agreeable man, who is a hypnotist of great experience, and enormous patience, and who has had most extraordinary results in some very inveterate cases, would be a promising man to consult” (Ibid., vol. 7, p. 397).

57 Hübbe to Ludwig Deinhard, November 17, 1906 (MS. Cod. Hübbe-Schleiden 65.2/16). Hugo Münsterberg continued his fight against psychical research in America. To bolster the usefulness of psychology as an applied science in the battle against the “social problem” of superstition, he staged an “exposure” of the famous medium Eusapia Palladino in New York (Münsterberg, 1910; Sommer, 2012a). On related actions by psychologists in the United States, see, for example, Coon (1992).

58 In 1917, Dessoir would send Wundt a copy of the first edition of his *Vom Jenseits der Seele* (“On the hereafter of the soul”), a critical popular overview of psychical research and occultism. An accompanying letter and a second letter in 1918 (Dessoir to Wundt, May 13, 1917, and January 22, 1918, Wundt papers, 1106/1–3 and 1107/1–3, University of Leipzig) confirm a statement Dessoir later made in his memoirs, namely, that his involvement in psychical research had served a “double intent to weed superstition and to win and free from its pre-forms the genuine values of belief and wisdom” (Dessoir, 1947a, p. 38). While the present paper is primarily concerned with political factors behind the repudiation of psychical research from nascent psychology, I am currently investigating a related topic, that is, the role of religious motivations in opponents of psychical research such as Wundt, Dessoir, Stanley G. Hall, and others.

59 After the schism in the MPG in 1889, journalists in Munich sometimes referred to members of du Prel's psychological society as “die Geprelten” (malapropism of “die Geprellten,” i.e., “the duped ones”), and to those of Schrenck's as “die Beschrenckten” (malapropism of “die Beschränkten,” i.e, “the dim ones”; Tischner, 1960, p. 204).

60 Also, Goeler von Ravensburg did not seem to have been subject to the “will to conform.” Economically independent and not intending to pursue an academic career, Goeler continued to argue, for example, in an article describing his participation in some of Richet's experiments in clairvoyance, for the reality of supernormal phenomena (Goeler von Ravensburg, 1890). Likewise, von Hartmann, who like du Prel was a private scholar, never ceased to propose, for example, clairvoyance as a natural phenomena to bolster his philosophy in the tradition of Hegel and Schopenhauer.

61 Du Prel to Aksakov, March 10, 1893, Pushkinskij Dom, St. Petersburg (not indexed).

## References

[b1] Alcock JE (1981). Parapsychology: Science or magic? A psychological perspective.

[b2] Alvarado CS (2002). Dissociation in Britain during the late nineteenth century: the Society for Psychical Research, 1882–1900. Journal of Trauma & Dissociation.

[b3] Bastian A (1887). Spiritismus und Ethnologie. Sphinx.

[b4] Bastian A (1890). Über psychische Beobachtungen bei Naturvölkern (Schriften der Gesellschaft für Experimental-Psychologie zu Berlin, II. Stück).

[b5] Bentivegni AV (1890). Die Hypnose und ihre civilrechtliche Bedeutung (Schriften der Gesellschaft für Experimental-Psychologie zu Berlin, IV. Stück).

[b6] Bérillon E (1892). Les applications de la suggestion hypnotique à l’éducation. In International Congress of Experimental Psychology. Second Session. London, 1892 (pp. 166–168).

[b7] Bernheim H (1887). Die hypnotische Suggestion, im Hinblick auf die Pädagogik betrachtet. Sphinx.

[b8] Bernheim H (1891). Suggestion und Psychotherapie. Sphinx.

[b9] Bleuler E (1929). Einige Gedenkworte an Schrenck-Notzing. Zeitschrift für Parapsychologie.

[b10] Bleuler E (1930). Vom Okkultismus und seinen Kritiken. Zeitschrift für Parapsychologie.

[b11] Braid J (1846). The power of the mind over the body, an experimental inquiry into the nature and cause of the phenomena attributed by Baron Reichenbach and others to a “new imponderable”.

[b12] Brandon R (1983). The spiritualists: The passion for the occult in the nineteenth and twentieth centuries.

[b13] Bringmann WG, Bringmann NJ, Ungerer GA, Bringmann WG, Tweney RD (1980). The establishment of Wundt's laboratory: An archival and documentary study. Wundt studies. A centennial collection.

[b14] Brower MB (2010). Unruly spirits. The science of psychic phenomena in modern France.

[b15] Brunn L (1889). Der Prophet. Sphinx.

[b16] Coon DJ (1992). Testing the limits of sense and science. American experimental psychologists combat spiritualism. American Psychologist.

[b17] Crabtree A (1993). From Mesmer to Freud. Magnetic sleep and the roots of psychological healing.

[b18] Crabtree A (2003). Automatism” and the emergence of dynamic psychiatry. Journal of the History of the Behavioral Sciences.

[b19] Dessoir M (1886a). Experimentelle Untersuchungen. Sphinx.

[b20] Dessoir M (1886b). Experiments in muscle-reading and thought-transference. Proceedings of the Society for Psychical Research.

[b21] Dessoir M (1886c). Gedanken-Übertragung. Ein Protokoll. Sphinx.

[b22] Dessoir M (1886d). Zur Geschichte des Gedankenlesens. Sphinx.

[b23] Dessoir M (1887a). Der Hypnotismus in Frankreich. Sphinx.

[b24] Dessoir M (1887b). Die Telepathie. Die Gegenwart.

[b25] Dessoir M (1887c). Hypnotism in France. Science.

[b26] Dessoir M (1887d). Verzeichnis der neueren Litteratur über Hypnotismus und verwandte Erscheinungen. Zu dem Aufsatze: Der Hypnotismus in Frankreich. Sphinx.

[b27] Dessoir M (1888a). Bibliographie des modernen Hypnotismus.

[b28] Dessoir M (1888b). Edmund Gurney zum persönlichen Gedächtnis. Sphinx.

[b29] Dessoir M (1888c). Ein Beitrag zur therapeutischen Verwerthung des Hypnotismus. Von Albert von Schrenck-Notzing. Psychische Studien.

[b30] Dessoir M (1888d). Neues über die Suggestion. Einige Bemerkungen. Sphinx.

[b31] Dessoir M (1889a). Der Hypnotismus auf der Berliner Hochschule. Sphinx.

[b32] Dessoir M (1889b). Die Parapsychologie. Eine Entgegnung auf den Artikel: “Der Prophet”. Sphinx.

[b33] Dessoir M (1889c). Über Arbeitsgebiet und Forschungsweise psychologischer Gesellschaften. Beilage zur Allgemeinen Zeitung.

[b34] Dessoir M (1890a). Aus dem Magazin für Erfahrungsseelenkunde. Sphinx.

[b35] Dessoir M (1890b). Das Doppel-Ich (Schriften der Gesellschaft für Experimental-Psychologie zu Berlin, I. Stück).

[b36] Dessoir M (1890c). Le double moi. Congrès International de Psychologie Physiologique. Pemière Session.

[b37] Dessoir M (1891). Experimentelle Pathopsychologie. Vierteljahrsschrift für wissenschaftliche Philosophie.

[b38] Dessoir M (1892). Hypnotismus und Suggestion. Von W. Wundt. Zeitschrift für Hypnotismus.

[b39] Dessoir M (1894). Geschichte der Neueren Deutschen Psychologie (2 vols.).

[b40] Dessoir M (1911). Abriß einer Geschichte der Psychologie.

[b41] Dessoir M (1912). Outlines of the history of psychology (D. Fisher, Trans.).

[b42] Dessoir M (1931). Vom Jenseits der Seele. Die Geheimwissenschaften in kritischer Betrachtung (6th rev. ed.).

[b43] Dessoir M (1947a). Buch der Erinnerung.

[b44] Dessoir M (1947b). Das Ich, Der Traum, Der Tod.

[b46] du Prel C (1869). Oneirokritikon. Der Traum vom Standpunkt des transzendentalen Idealismus. Deutsche Vierteljahrs-Schrift.

[b47] du Prel C (1870). Dr. E. von Hartmanns Philosophie des Unbewußten. Deutsche Vierteljahrs-Schrift.

[b48] du Prel C (1872a). Der gesunde Menschenverstand vor den Problemen der Wissenschaft. In Sachen J. C. Fischer contra Eduard von Hartmann.

[b49] du Prel C (1872b). Philosophie des Unbewußten. Von Eduard von Hartmann [review, 3rd ed. of Hartmann, 1869]. Oesterreichische Wochenschrift für Wissenschaft und Kunst.

[b50] du Prel C (1880). Die Planetenbewohner und die Nebularhypothese. Neue Studien zur Entwicklungsgeschichte des Weltalls.

[b51] du Prel C (1881). Der Aberglaube und die Wissenschaft. Der Salon, II.

[b52] du Prel C (1882a). Sind Träume Schäume?. Kosmos.

[b53] du Prel C (1882b). Ueber die Entwicklungsfähigkeit der Wissenschaft. Kosmos.

[b54] du Prel C (1882c). Ueber die wissenschaftliche Bedeutung des Traumes. Kosmos.

[b55] du Prel C (1884). Der Somnambulismus. Die Gegenwart.

[b56] du Prel C (1885a). Die Philosophie der Mystik.

[b57] du Prel C (1885b). Professor Preyer über das Gedankenlesen. Die Gegenwart.

[b58] du Prel C (1886). Monistische Seelenlehre. Sphinx.

[b59] du Prel C (1887). Programm der psychologischen Gesellschaft in München. Sphinx.

[b60] du Prel C (1888). Die monistische Seelenlehre. Ein Beitrag zur Lösung des Menschenrätsels.

[b61] du Prel C (1889a). Das hypnotische Verbrechen und seine Entdeckung.

[b62] du Prel C (1889b). Die Mystik im Irrsinn. Psychische Studien.

[b63] du Prel C (1890). Studien aus dem Gebiet der Geheimwissenschaften (2 vols.).

[b64] du Prel C (1894). Die Entdeckung der Seele durch die Geheimwissenschaften (2 vols.).

[b65] du Prel C (1899). Die Magie als Naturwissenschaft (2 vols.).

[b66] du Prel C (1911). Nachgelassene Schriften.

[b67] du Prel C, du Prel C (1889). Die psychologischen Gesellschaften. Nachgelassene Schriften.

[b68] Ellenberger HF (1970). The discovery of the unconscious: The history and evolution of dynamic psychiatry.

[b69] Forel A (1891). Ein Gutachten über einen Fall von spontanem Somnambulismus mit angeblicher Wahrsagerei und Hellseherei (Schriften der Gesellschaft für psychologische Forschung, Heft I).

[b70] Forel A (1894). Durch Spiritismus erkrankt und durch Hypnotismus geheilt. Zeitschrift für Hypnotismus.

[b71] Freytag N, de Blécourt W, Davies O (2004). Witchcraft, witch doctors and the fight against “superstition” in nineteenth-century Germany. Witchcraft continued. Popular magic in modern Europe.

[b72] Gauld A (1968). The founders of psychical research.

[b73] Gauld A (1992). A history of hypnotism.

[b179] Goeler von Ravensburg FK (1890). Das Hellsehen.

[b74] Goeler von Ravensburg FK, Dessoir M (1888). Programm der Gesellschaft für Experimental-Psychologie zu Berlin. Sphinx.

[b75] Grashey H, Hirt L, von Schrenck-Notzing A, Preyer WT (1895). Der Prozeß Czynski. Thatbestand desselben und Gutachten ueber Willensbeschränkung durch hypnotisch-suggestiven Einfluß, abgegeben vor dem oberbayerischen Schwurgericht zu München.

[b76] Gurney E (1887). Telepathie: Eine Erwiderung auf die Kritik des Herrn Prof. W. Preyer.

[b77] Hale M (1980). Human science and social order: Hugo Münsterberg and the origins of applied psychology.

[b78] Hall TH (1962). The spiritualists: The story of Florence Cook and William Crookes.

[b79] Hamilton T (2009). Immortal longings: F.W.H. Myers and the Victorian search for life after death.

[b80] Hansen FCC, Lehmann A (1895). Ueber unwillkürliches Flüstern. Eine kritische und experimentelle Untersuchung der sogenannten Gedankenübertragung. Philosophische Studien.

[b85] Hövelmann GH

[b86] Hübbe-Schleiden W (1886). Aufruf und Vorwort. Sphinx.

[b87] Hübbe-Schleiden W (1889a). Hypnotische Sitzung der Psycholog. Gesellschaft in München. Sphinx.

[b88] Hübbe-Schleiden W (1889b). Internationaler Kongreß der Société de psychologie physiologique. Sphinx.

[b89] Imkoff F (1889). Das hypnotische Verbrechen und seine Entdeckung (Rezension). Sphinx.

[b90] Imkoff F (1890). Die gerichtliche Bedeutung und mißbräuchliche Anwendung des Hypnotismus. Sphinx.

[b91] Imkoff F (1891a). Der Hypnotismus und seine Handhabung. Mit besonderer Berücksichtigung des Mesmerismus und der Schulwissenschaft. Sphinx.

[b92] Imkoff F (1891b). Klinische Vorlesungen über Hypnotismus. Sphinx.

[b93] James W (1894). Review of “Cock-Lane and Common Sense” by Andrew Lang, and “Die Entdeckung der Seele durch die Geheimwissenschaften” by Carl du Prel. Psychological Review.

[b94] Kaiser T (2008).

[b95] Kelly EF, Kelly EW, Crabtree A, Gauld A, Grosso M, Greyson B (2007). Irreducible mind. Toward a psychology for the 21st century.

[b97] Krohn WO (1892). Facilities in experimental psychology at the various German universities. American Journal of Psychology.

[b98] Kurzweg A (1976).

[b99] Liébeault A-A (1888). Geständnisse eines ärztlichen Hypnotisten. Sphinx.

[b100] Liébeault A-A (1891). Über das Hellsehen. Sphinx.

[b101] Lipps T (1897). Raumästhetik und geometrisch-optische Täuschungen (Schriften der Gesellschaft für Psychologische Forschung. Heft IX/X).

[b102] Lipps T (1902). Vom Fühlen, Wollen und Denken (Schriften der Gesellschaft für Psychologische Forschung, Heft 13 u. 14).

[b103] Lombroso C (1909). After Death—What? Spiritistic phenomena and their interpretation (W. S. Kennedy, Trans.).

[b104] Mauskopf SH, McVaugh MR (1980). The elusive science. Origins of experimental psychical research.

[b105] Moll A (1892). Der Rapport in der Hypnose. Untersuchungen über den thierischen Magnetismus (Schriften der Gesellschaft für psychologische Forschung, Heft 4).

[b106] Moll A (1893). Die conträre Sexualempfindung. Mit Benutzung amtlichen Materials (2nd enlarged ed.).

[b107] Moll A (1903). Spiritistische Wahngebilde. Zeitschrift für Pädagogische Psychologie, Pathologie und Hygiene.

[b108] Möller P (1905). Die Bedeutung des Urteils für die Auffassung (Schriften der Gesellschaft für Psychologische Forschung, Heft 15).

[b109] Münsterberg H (1889). Gedankenübertragung. Vortrag gehalten in der Akademischen Gesellschaft zu Freiburg i. B. am 10. Januar 1889.

[b110] Münsterberg H (1891). Ueber die Aufgaben und Methoden der Psychologie (Schriften der Gesellschaft für psychologische Forschung, Heft 2).

[b111] Münsterberg H, Münsterberg H (1892). Zeitausfüllung. Beiträge zur experimentellen Psychologie, Heft 4.

[b112] Münsterberg H (1910). My friends the spiritualists: Some theories and conclusions concerning Eusapia Palladino. Metropolitan Magazine.

[b113] Myers FWH (1887). Die menschliche Persönlichkeit im Lichte der hypnotischen Eingebung. Sphinx.

[b115] Myers FWH (1893). Professor Wundt on hypnotism and suggestion. Mind.

[b116] Nahm M (2012). The sorcerer of Cobenzl and his legacy: The life of baron Karl Ludwig von Reichenbach, his work and its aftermath. Journal of Scientific Exploration.

[b117] Notes (1888). American Journal of Psychology.

[b118] Offner M (1891). Die deutsche Gesellschaft für psychologische Forschung. Sphinx.

[b119] Oppenheim J (1985). The other world. Spiritualism and psychical research in England, 1850–1914.

[b120] Osier DV, Wozniak RH (1984). A century of serial publications in psychology, 1850–1959. An international bibliography.

[b121] Parish E (1894). Ueber die Trugwahrnehmung (Hallucination und Illusion) mit besonderer Berücksichtigung der internationalen Enquête über Wachhallucinationen bei Gesunden (Schriften der Gesellschaft für psychologische Forschung, Heft 7/8).

[b122] Plas R (2000). Naissance d'une Science Humaine: La Psychologie. Les Psychologues et le “Merveilleux Psychique”.

[b123] Preyer WT (1878). Der thierische Magnetismus und der Mediumismus einst und jetzt. Deutsche Rundschau.

[b124] Preyer WT (1886a). Die Erklärung des Gedankenlesens nebst Beschreibung eines neuen Verfahrens zum Nachweise unwillkürlicher Bewegungen.

[b125] Preyer WT (1886b). Telepathie und Geisterseherei in England. Deutsche Rundschau.

[b126] Rells (1891). Dr. von Schrencks neueste Schriften. Sphinx.

[b127] Richet C (1891). Erscheinungen Lebender. Sphinx.

[b150] Shamdasani S (1993). Automatic writing and the discovery of the unconscious. Spring.

[b151] Shamdasani S, Shamdasani S (1994). Encountering Hélène: Théodore Flournoy and the genesis of subliminal psychology. Théodore Flournoy. From India to the planet Mars: A case of multiple personality with imaginary languages.

[b152] Sidgwick EM, Sidgwick A (1906). Henry Sidgwick. A memoir.

[b153] Sidgwick H (1892). Presidential address. In International Congress of Experimental Psychology. Second Session. London, 1892.

[b154] Sidgwick H (1896). Involuntary whispering considered in relation to experiments in thought-transference. Proceedings of the Society for Psychical Research.

[b155] Sidgwick H (1897). Experiments in involuntary whispering, and their bearing on alleged cases of thought-transference. Dritter Internationaler Congress für Psychologie.

[b156] Skrupskelis IK, Berkeley EM (1992). The correspondence of William James (12 vols.).

[b157] Sommer A (2009). From astronomy to transcendental Darwinism: Carl du Prel (1839–1899). Journal of Scientific Exploration.

[b158] Sommer A (2011). Professional heresy: Edmund Gurney (1847–1888) and the study of hallucinations and hypnotism. Medical History.

[b159] Sommer A (2012a). Psychical research and the origins of American psychology: Hugo Münsterberg, William James and Eusapia Palladino. History of the Human Sciences.

[b160] Sommer A (2012b). Policing epistemic deviance: Albert von Schrenck-Notzing and Albert Moll. Medical History.

[b161] Stern WL (1900). Über Psychologie der individuellen Differenzen (Ideen zu einer “Differentiellen Psychologie”) (Schriften der Gesellschaft für Psychologische Forschung, Heft 11).

[b162] Stumpf C, Murchison C (1930). Autobiography. History of psychology in autobiography.

[b163] Sulloway FJ (1992). Freud, biologist of the mind: Beyond the psychoanalytic legend.

[b164] Taylor E (1983). William James on exceptional mental states. The 1896 Lowell Lectures.

[b165] Taylor E (1996). William James: On consciousness beyond the margin.

[b166] Tischner R (1960). Geschichte der Parapsychologie.

[b167] Treitel C (2004). A science for the soul. Occultism and the genesis of the German modern.

[b168] Vogt O, Maack F (1898). Antwort von Dr. Oskar Vogt. Okkultismus. Was ist er? Was will er? Wie erreicht er sein Ziel? Eine unparteiische Rundfrage.

[b169] Vogt O (1901). Contre le spiritisme [with discussion]. IV^E^ Congrès International de Psychologie. Tenu à Paris, du 20 au 26 Août 1900.

[b81] von Hartmann E (1869). Philosophie des Unbewußten. Versuch einer Weltanschauung.

[b82] von Hartmann E (1886). Moderne Probleme.

[b83] von Hellenbach L (1876). Eine Philosophie des gesunden Menschenverstandes. Gedanken über das Wesen der menschlichen Erscheinung.

[b84] von Hellenbach L (1885). Geburt und Tod als Wechsel der Anschauungsform oder die Doppel-Natur des Menschen.

[b96] von Krafft-Ebing R (1907). Psychopathia Sexualis. Mit besonderer Berücksichtigung der konträren Sexualempfindung. Eine medizinisch-gerichtliche Studie für Ärzte und Juristen. Herausgegeben von Dr. Alfred Fuchs (13th enlarged ed.).

[b128] [von Schrenck-]Notzing A (1886). Übersinnliche Willens-Übertragung mit und ohne Hypnose. Sphinx.

[b129] [von Schrenck-]Notzing A (1887a). Experimente übersinnlicher Eingebung, hypnotisch und posthypnotisch. Sphinx.

[b130] [von Schrenck-]Notzing A (1887b). Telepathische Experimente des Sonderausschusses der Psychologischen Gesellschaft zu München. Sphinx.

[b131] [von Schrenck-]Notzing A (1887c). Unmittelbare Willensübertragung. Sphinx.

[b132] [von Schrenck-]Notzing A (1887d). Warnende Wahrträume. Sphinx.

[b133] [von Schrenck-]Notzing A (1888a). Dessoirs Bibliographie. Sphinx.

[b134] [von Schrenck-]Notzing A (1888b). Fortschritte des Hypnotismus. Neuere Publikationen. Sphinx.

[b135] [von Schrenck-]Notzing A (1888c). Hypnotische Experimente. Comitébericht der psychologischen Gesellschaft in München. Psychische Studien.

[b136] [von Schrenck-]Notzing A (1889). Die Suggestionstherapie und ihre Technik. Sphinx.

[b137] von Schrenck-Notzing A (1888). Ein Beitrag zur therapeutischen Verwerthung des Hypnotismus.

[b138] von Schrenck-Notzing A (1889). Ueber die praktische Bedeutung des Hypnotismus: Vortrag, gehalten im Kaufmännischen Verein am 8. Oktober 1889.

[b139] von Schrenck-Notzing A (1890a). Association des sociétès de psychologie. Congrès International de Psychologie Physiologique. Pemière Session [Paris: 1889].

[b140] von Schrenck-Notzing A (1890b). Photographies d'hypnotiques. Congrès International de Psychologie Physiologique. Pemière Session.

[b141] von Schrenck-Notzing A (1891a). Experimental studies in thought-transference. Proceedings of the Society for Psychical Research.

[b142] von Schrenck-Notzing A, Richet C (1891b). Vorrede des Uebersetzers. Experimentelle Studien auf dem Gebiete der Gedankenübertragung und des sogenannten Hellsehens.

[b143] von Schrenck-Notzing A (1891c). Ein schwerer sensitiv-somnambuler Krankheitsfall geheilt ausschliesslich mittelst einfacher Anwendung der Gesetze des Odes. Für Physiker, Physiologen, Psychologen, Psychiatriker und insbesondere für Ärzte. Von Karl Freiherr von Reichenbach.

[b144] von Schrenck-Notzing A (1892). Die Suggestions-Therapie bei krankhaften Erscheinungen des Geschlechtssinns, mit besonderer Berücksichtigung der conträren Sexualempfindung.

[b145] von Schrenck-Notzing A (1893). Ueber Suggestion und suggestive Zustände (Vortrag in der Anthropologischen Gesellschaft, München, 17. März 1893).

[b146] von Schrenck-Notzing A (1895a). Ein Beitrag zur Aetiologie der conträren Sexualempfindung.

[b147] von Schrenck-Notzing A (1895b). Ueber unwillkürliches Flüstern. Eine kritische und experimentelle Untersuchung der sogenannten Gedankenübertragung. Von F. C. C. Hansen und Alfred Lehmann. Wundt's Philosoph. Studien. Bd. 11 [review]. Zeitschrift für Hypnotismus.

[b148] von Schrenck-Notzing A (1904). Die Traumtänzerin Magdeleine G. Eine psychologische Studie über Hypnose und dramatische Kunst. Unter Mitwirkung des F.E. Otto Schultze.

[b149] von Schrenck-Notzing A (1914). Materialisations-Phaenomene. Ein Beitrag zur Erforschung der mediumistischen Teleplastie.

[b170] Walther G, Walther G (1962). Chronologisches Verzeichnis der Veröffentlichungen des Dr. med. A. Freiherrn von Schrenck-Notzing. Dr. med. Albert Freiherr von Schrenck-Notzing. Grundfragen der Parapsychologie.

[b171] Williams JP (1984). The making of Victorian psychical research: An intellectual elite's approach to the spiritual world.

[b172] Wolffram H (2006). Parapsychology on the couch: the psychology of occult belief in Germany, c. 1870–1939. Journal of the History of the Behavioral Sciences.

[b173] Wolffram H (2009). Stepchildren of science: Psychical research and parapsychology in Germany, c. 1870–1939 (Clio Medica: The Wellcome Series in the History of Medicine).

[b174] Wreschner A (1898). Methodologische Beiträge zu psychophysiologischen Messungen (auf experimenteller Grundlage) (Schriften der Gesellschaft für Psychologische Forschung, Heft 11).

[b175] Wundt W (1879). Der Spiritismus. Eine sogenannte wissenschaftliche Frage. Offener Brief an Herrn Prof. Dr. Hermann Ulrici in Halle.

[b176] Wundt W (1892). Hypnotismus und Suggestion.

[b177] Wundt W (1894). Lectures on Human and Animal Psychology (J. E. Creighton & E. B. Titchener, Trans.).

[b178] Zwikirsch B (1994). Der Nachlaß “Max Dessoir” im Preußischen Geheimen Staatsarchiv in Berlin-Dahlem. Ein Beitrag zur Geschichte der Psychologie in Berlin. Psychologie und Geschichte.

